# Proceedings of Rochester Hahnemannian Society

**Published:** 1888-09

**Authors:** W. H. Baker

**Affiliations:** Secretary


### PROCEEDINGS OF THE ROCHESTER HAHNEMANN SOCIETY.

The regular monthly meeting of the Rochester Hahnemann Society was called to order at the office of Dr. J. A. Biegler, by the President, Dr. Grant.

Members present, Drs. Grant, Schmitt, Biegler, Carr, Hoard, and Baker. Dr. Brownell, of this city, was present as a visitor.

Sections 146 to 156 of the *Organon* were read by the Secretary, with the following discussion :

Dr. Biegler—One of the best lessons in the section read is the injunction to grasp the peculiar symptoms. Often in cases where we get such general indications as fatigue, sleeplessness, etc., we will find among them some peculiar symptom. Every day I meet peculiar symptoms; for instance, a peculiar symptom would be thirst during chill. Unless you have your mind on the case you will miss them.

Dr. Schmitt—I would like Dr. Brownell to give us notes of a case he treated which I think illustrates this point.

Dr. Brownell—It is a case of acute articular rheumatism, in a man of light hair and eyes, and a peculiar red face. I do not remember all the symptoms, but will give what I can. This man was taken sick with pain in every joint, very severe and ugly, and very restless. Rhus tox.$^{cm}$, one dose; not much improved under this remedy. After this he received Bry.$^{cm}$, one dose; then I noticed *he kept trying to brush a hair off of his face;* this was very marked; he had a toothbrush tied on a stick so he might use it to brush his face. I mentioned the case to Dr. Grant, and he recommended Graphites. I also looked the case up with Dr. Schmitt, and we found Borax, Phos-ac., Baryta, Ran-scel., and Graphites had the symptom, but Graphites covered the case. One dose of the CM cured the case in twenty-four hours.

Dr. Schmitt—A short time ago I had a patient who had a peculiar symptom that I have verified a number of times, but cannot tell where I found it. The case was that of a child four years of age, that an allopath had been treating for diphtheria. I found the child under the influence of Opium, fever, delirium, jerked in its sleep, vomiting. I looked into the throat, but could not see any membrane. Diagnosis, unknown disease, mixed up with allopathic drugs, was what I told the mother. Belladonna was given; the chief reason it being an antidote to Opium. After the dose the child slept at night; next day better. Fever came on at eleven A. M.; gave another dose, as I thought one was not enough to antidote the large amount of Opium the child had received, and left a second dose to give if not better in the night; the next day the fever came on at eleven A. M. again, then this peculiar symptom came up, *she wanted to sleep with her hands up over the head*, which the mother informed me was new for her; there was also thirst for small quantities and often; involuntary stool, like chocolate. Ars.$^{cm}$ finished the case. Verat-alb. has this symptom also.

Dr. Biegler—I do not like to give a remedy before a sickness of any kind, as the menses, etc. I wait until all is over, then give my remedy. If I know of a fever coming on I wait until it is past. I believe this is the best plan, for you get less aggravation, and you will oftener get through with one dose if you wait until after the aggravation.

Dr. Schmitt—I often give Belladonna in the afternoon.

Dr. Biegler—If I cannot give Belladonna in the morning, I wait until after four P. M.

Dr. V. A. Hoard, the essayist of the evening, read his paper:

GENTLEMEN:—I will report a case illustrating the efficiency of potentized remedies in opium eaters.

Mrs. M——, aged sixty-one years, has always been in poor health. Twenty years ago an old-school physician prescribed Morphine for dysmenorrhœa, this was the beginning of the habit of which she is now a victim, and is taking one drachm of Morphine a week, or about eight and one-half grains per day. She was taken, in the evening, with a sudden attack of what she called an indescribable pain in the pit of the stomach, which radiated from there all over the body. Unquenchable thirst; water is vomited as soon as reaching the stomach. Paroxysms of violent retching and vomiting. Vertigo, prostration; small, weak pulse; cold sweat on face; body cold and dry; features drawn and all the symptoms of collapse; bowels undisturbed at first, but after two days a painter's diarrhœa; mouth very sore and great soreness and burning in the epigastric region.

In prescribing, the question arose in my mind, whether a higher potency would act beneficially, or would it be necessary to give the crude drug. It was evident that some active measure should be taken at once.

She was taking such enormous quantities of Morphine daily, that palliation by that was out of the question, even had I been so disposed. I gave her Arsenicum$^{30}$, called the next morning and found no improvement; then changed to Phos., called again in the evening and found her condition unchanged; then gave her Bismuth$^{30}$. After the second powder the vomiting became less frequent, and when I saw her the next morning had almost ceased, and the pain had changed to a dull ache which disappeared by the third day. It was with considerable hesitation that I prescribed as I did, but on thinking of the successful prescriptions that are made, daily, to persons who are accustomed to the use of some drug, as tea, coffee, cocoa, or more particularly tobacco, and that the primary action of all of these, including Morphine, is diminished by their habitual use, it would seem that nature adapts herself to this abnormal condition for a time, and the functions of the body are performed in a seemingly normal manner, but sooner or later nature asserts herself, and the nerves, which before have been soothed and quieted, become irritable and render life miserable. Such I believe to have been the case with this woman. Professor T. F. Allen, in a series of lectures on the above-mentioned drugs, said that he believed that many of the potentized remedies would act beneficially when prepared in coffee or given to persons who were in the habit of using it. In speaking of tobacco, he reported a case of seasickness cured by Tobacco$^{200}$ in a person who had been an inveterate smoker for years. Another point illustrated in this case, is that Homœopathy as a school and we as physicians, can never be held accountable for causing a habit which brings so much of suffering and misery.

Discussion.—Dr. Biegler—Did she get over the Morphine habit?

Dr. Hoard—No.

Dr. Schmitt—Did she have the cold sweat on the face when you gave Bismuth?

Dr. Hoard—Yes.

Dr. Schmitt—It is new to me that Bismuth has that symptom.

Dr. Grant—I had a case from allopathic hands, was sent for at nine P. M., found a case of gall-stone colic. The physician in charge had given Morphine for some hours without relief. They sent to druggist for one-quarter grain pills of Morphine, one every half hour; grew worse; no sleep or thought of sleep; *there was a cutting pain, shooting up into the chest; had awakened first at four* A. M. *with the pain; at four* P. M. *was decidedly worse; swing-like motion of alæ nasi.* Lyc.$^{2c}$, one dose, and S. L.

Next morning quiet, better, but sore and weak. In fifteen minutes after the dose of Lycopodium the pain was gone, and before the next dose of Sac. lac. went to sleep. There is a question in the case whether the stone passed and relieved the pain or that the remedy should receive the credit, or that whether the Morphine had anything to do with the relief.

Dr. Biegler—I will give you the case of a lady I saw last Sunday morning, and there was no Morphine used in this case.

It is the case of renal colic in a lady who has a chronic nephritis, a bad subject to relieve. I saw her first about eleven A. M. not expecting to find very much the matter with her, but as I went into the house, I could hear her cry from the intense pain, that I learned she had suffered with for four hours. She was running about the room, could not keep still. It was a hot day, but she told me to keep the door shut, could not bear cold air, and the room was very hot and close. Her son, a recent graduate of the University of Pennsylvania, arrived home that morning. His diagnosis was suppressed menses. I told him to never mind the name at present. We should have three legs to our stool when we prescribe. Here we had—*Severe pain in region of kidney and bladder; desire to urinate, but could not; desire to eructate gas, but could not; pain on right side and worse from cold air.* Lyc.$^{mm}$, one dose on the tongue; in fifteen to twenty minutes felt better; in one-half hour was running about again; did nothing, but allowed the son to give Sac. lac. often. In one hour after the dose, I informed her I wished to make a local examination. My object was to see if she could lie down. In the meantime she began to eructate, and urinated; was satisfied to lie down on right side, could not on the left. I then dissolved a dose of Lyc. in four teaspoonfuls of water, one teaspoonful to be given in two or three hours if pain returned.

Her son reported in the afternoon, that just after I left she had a spasm, which lasted about ten minutes. After this she grew better, and to-day is quiet and doing well on the one dose.

Moved and seconded this paper be accepted and published in THE HOMŒOPATHIC PHYSICIAN.

Dr. Carr was appointed essayist.

Adjourned to Dr. Schmitt's office, third Tuesday in July.

W. H. BAKER, *Secretary.*

